# Diversity in conserved genes in tomato

**DOI:** 10.1186/1471-2164-8-465

**Published:** 2007-12-18

**Authors:** Allen Van Deynze, Kevin Stoffel, C Robin Buell, Alexander Kozik, Jia Liu, Esther van der Knaap, David Francis

**Affiliations:** 1 Seed Biotechnology Center, University of California, 1 Shields Ave., Davis, CA, USA; 2 The Institute for Genomic Research, 9712 Medical Center Dr, Rockville, MD, USA; 3 Department of Plant Biology, Michigan State University, 166 Plant Biology, East Lansing, MI, USA; 4 Genome and Biomedical Sciences Facility, University of California, 1 Shields Ave., Davis, CA, USA; 5 Department of Horticulture and Crop Science, The Ohio State University/OARDC, 1680 Madison Ave, Wooster, OH, USA

## Abstract

**Background:**

Tomato has excellent genetic and genomic resources including a broad set of Expressed Sequence Tag (EST) data and high-density genetic maps. In addition, emerging physical maps and bacterial artificial clone sequence data serve as template to investigate genetic variation within the cultivated germplasm pool with the goal to manipulate agriculturally important traits. Unfortunately, the nearly exclusive focus of resource development on interspecific populations for genetic analyses and diversity studies has left a void in our understanding of genotypic variation within tomato breeding programs that focus on intra-specific populations. We describe the results of a study to identify nucleotide variation within tomato breeding germplasm and mapping parents for a set of conserved single-copy ESTs that are orthologous between tomato and *Arabidopsis*.

**Results:**

Using a pooled sequencing strategy, 967 tomato transcripts were screened for polymorphism in 12 tomato lines. Although intron position was conserved, intron lengths were 2-fold larger in tomato than in *Arabidopsis*. A total of 1,487 single nucleotide polymorphisms and 282 insertion/deletions were identified, of which 579 and 206 were polymorphic in breeding germplasm, respectively. Fresh market and processing germplasm were clearly divergent, as were *Solanum lycopersicum var. cerasiformae* and *Solanum pimpinellifolium*, tomato's closest relatives. The polymorphisms identified serve as marker resources for tomato. The COS is also applicable to other Solanaceae crops.

**Conclusions:**

The results from this research enabled significant progress towards bridging the gap between genetic and genomic resources developed for populations derived from wide crosses and those applicable to intra-specific crosses for breeding in tomato.

## Background

Tomato (*Solanum lycopersicum, Sl*) has rich genetic and genomic resources including comprehensive databases of Expressed Sequence Tags (ESTs), Bacterial Artificial Chromosome (BAC) libraries, and genetic and comparative maps which are in the process of being linked to a physical map and eventually the euchromatic genomic sequence. These resources serve as template to study genetic variation and to manipulate agricultural traits. Current genetic maps for tomato include 2,200 Restriction Fragment Length Polymorphisms (RFLPs), Cleaved Amplified Polymorphic Sequences (CAPs), and Simple Sequence Repeats (SSRs), as well as emerging genetic resources which include a comparative map with *Arabidopsis *of over 500 Conserved Orthologous Set (COS) markers [1,2]. These maps were derived from populations that were developed between wild relatives (various *Solanum *species) and cultivated varieties. This approach maximizes genetic variation and has led to the discovery and introgression of novel alleles for disease resistance [3]and fruit traits [4,5] into cultivated germplasm. However, the nearly exclusive focus on wide crosses has left a void in our knowledge and ability to manipulate other traits of agricultural importance within cultivated tomato. There is a lack of molecular markers that detect nucleotide polymorphisms among elite breeding lines. With the exception of genes that were introgressed from wild species, the majority of the breeding efforts in tomato are derived from elite-by-elite intra-specific crosses, resulting in consistent improvement for yield and fruit quality [6]. Increasing molecular marker density to facilitate the evaluation and analysis of elite-by-elite breeding populations is highly desirable.

Given the discrepancy of resources available between inter-specific and intra-specific crosses, we sought to test the feasibility of a large-scale screen for identifying polymorphisms within cultivated tomato lines. Previous examinations of EST databases suggested that Single Nucleotide Polymorphisms (SNPs) could be detected within tomato EST databases [7,8]. Single nucleotide polymorphisms, are highly abundant in plant species. They can be associated with known genomic sequences and are bi-allelic [9]. Current technology allows SNPs to be assayed cost-effectively for applications in breeding programs and genetic studies. Analysis of tomato EST sequences resulted in only limited information on SNPs that occurred within cultivated tomato germplasm. In a previous study, the GenBank EST database was mined for SNPs which resulted in 101 putative SNPs, 83 of which were confirmed empirically. Of the 83 validated SNPs, 53% proved useful within cultivated germplasm [8]. A similar study using an algorithm that considered sequence context, but not sequence redundancy, identified 764 putative SNPs from a similar dataset from which only 27% (28/103) of the putative SNPs tested could be validated experimentally [7]. The paucity of SNPs within the ESTs can be attributed to low rate of polymorphism within gene coding regions of tomato and a limited sampling of genotypes in the databases, and shallow sampling within each of these genotypes. The current resources are thus insufficient for breeding studies.

Extrapolation of these experimentally defined SNP rates to the gene and genome level projects a rate of 1 SNP every 4000–8500 bp of coding sequence. Variation in genes has been shown to be highest in untranslated regions (UTRs) [9] and within introns in which nucleotide diversity is more tolerated compared to protein coding regions [10]. Often, intron position is conserved between species [11], therefore one can leverage the *Arabidopsis *genome sequence and orthologous EST sequences from different species to predict intron position. Thus, a strategy for SNP discovery is to utilize available EST sequences and design primers that flank introns or amplify the 3' or 5' UTR from genomic DNA.

Genomic resources of *Arabidopsis *can be used to identify single-copy genes in tomato which in turn can be used as template to develop DNA markers. A COS is defined as a set of genes that are conserved throughout plant evolution in both sequence and copy number [12,13]. Sequences from a single-copy COS of *Arabidopsis *can be used as a reference to bridge relationships across genes identified through ESTs in crops. A COS in tomato (SGN COS I) was identified by comparing the set of unique gene sequences in tomato (*Sl *only) to all translated proteins in the fully sequenced genome of *Arabidopsis *[12,14]. Approximately 10% of the 27,000 tomato unigene sequences meet the defined COS criteria. The SGN COS I set was further refined to include pepper, potato, eggplant and coffee for a combined COS (SGN COS II) of 2,869 unigenes [14] across five species. Southern hybridization revealed that 85% of the COS tested are single-copy sequences in tomato and 13 other plant species from the Solanaceae, Asteraceae, Leguminosae, Cucurbitaceae, Rosaceae, Brassicaceae, Poaceae, and Malvaceae families [12]. Using different approaches, we identified 2,185 sequences as a COS between *Arabidopsis *and tomato, lettuce, sunflower, soybean and maize, of which 1,704 are represented in tomato. The parameters for developing the latter dataset are described at Compositae Genome Project Database [15] and summarized in Results and Discussion. A COS database provides a unique resource that can be applied to marker development for crop improvement and assist in comparative genomic analyses. In this study, we developed primers flanking predicted introns in the tomato COS and used these to amplify genomic DNA from several tomato genotypes. Amplified loci were pre-screened to identify sequence variation using pools of DNA from diverse genotypes. Loci identified as polymorphic were subsequently sequenced for 12 genotypes representing closely related wild relatives, processing tomato germplasm adapted to arid and humid growing conditions, fresh-market germplasm, and heirloom germplasm. The resulting sequence data, described in the current paper, is intended to greatly increase the availability of DNA-based markers for intra-specific tomato crosses and to characterize and manipulate traits important for tomato breeding populations.

## Results and Discussion

### Conserved Orthologous Sets

Our goal is to identify and characterize sequence variation within cultivated tomato in genes that can be used in genetic analysis, functional genomic studies, and breeding efforts. By focusing on a COS as the basis for marker discovery, we also intended to develop a sequence resource representing single-copy loci that can be assayed reproducibly for genetic analysis within *Sl *and in other related species. The challenge to identify a COS with incomplete EST datasets is to accurately predict single-copy and orthologous genes that do not represent paralogs. EST databases are highly redundant and unigene sets of assembled ESTs tend to over-represent the complement of transcripts. For example, both the *Arabidopsis *and Rice EST databases (> 700,000 ESTs) represent about 150,000 unigenes compared to less than 30,000–45,000 predicted genes with whole genome sequence [16]. The difference in number is attributable to allelic variation, sequencing errors, alternative splice forms, and paralogs which can not be easily addressed in the assembly of transcripts in the absence of a complete genome and sequence of full length cDNAs.

Designing a COS involves decisions regarding stringency of match, order of comparisons, and definitions of orthologs [14,17]. One approach taken to identify a COS is to compare unigene sets anchored to single-copy loci in *Arabidopsis *and generate reciprocal best matches for pairwise comparisons among several species and *Arabidopsis *[14]. With this strategy, only unigenes with a single best match to *Arabidopsis *are considered. In tomato, this approach resulted in a COS of 2,587 sequences (SGN COS II) which was further used to design primers with homology across species to amplify loci in tomato, pepper, potato and coffee. Fifteen loci were sequenced across these species for phylogenetic analysis. The strategy resulted in a COS that over-represented genes involved in organelle and chloroplast-associated proteins and DNA/RNA metabolism, and under-represented cell wall, transcription factors, protein kinases and signal transduction peptides [14]. We used a more conservative approach to identify a COS by first identifying single-copy proteins in *Arabidopsis *using a "BLASTP all-vs-all" search and then comparing single-copy *Arabidopsis *genes to all tomato ESTs (all *S. lycopersicum*, *Solanum habrochaites *and *Solanum pennellii *ESTs in GenBank in 2003) using TBLASTX. From these results, we identified the single best tomato EST match (usually the longest) to an *Arabidopsis *sequence and performed a BLASTN search against the *Arabidopsis *genome to verify the hit. The 1,704 tomato ESTs that had a single hit in *Arabidopsis *were included in a COS which we designate UCD COS to distinguish it from the SGN COS I and SGN COS II. As the number of ESTs used to create the UCD COS was less than that used in the SGN COS II created later we expect the UCD COS to have less sequences than SGN COSII. By selecting a single EST for each contig, incorrect assemblies of paralogs and errors due to alternative splicing are avoided [15].

For transparency, we cross-referenced all UCD Tomato COS to the SGN COS II integrated dataset for *Solanum *species (Table 1, See Additional file 1,[14]). The 1,704 UCD COS have single hits with a BLAST expect value cutoff of e-20 over 80% of the UCD contig length to 1,611 SGN Unigenes (SGN build Tomato200607,[1]), 857 tomato, 729 potato, 418 pepper and 442 coffee COS II markers. The UCD COS represents 847 unigenes that are not in SGN COS II which expands the total COS by 33% from 2,587 (SGN COS II) to 3,434 unigenes (See Additional file 1). To further investigate the application of the UCD COS, we evaluated the ability of 96 primers designed solely for tomato to amplify single-copy sequences in pepper and eggplant. Primers designed from loci with COS represented in at least two species and *Arabidopsis *were more likely to amplify single-copy loci in eggplant (58 vs. 38%) and pepper (44 vs. 25%) than those found only in the tomato COS. Only two primers in eggplant and four primers in pepper amplified multiple fragments (data not shown). This verifies the utility of conserved sequence in species with less genomic resources. Although the independent approaches to COS development are similar, we have shown that they verify each other, yet can be complementary. Nevertheless, both COS sets empirically yielded genomic resources to characterize putative single-copy conserved genes.

**Table 1:** Number of UCD COS in common with SGN COSII.

**Species**	**Total^1^**
Tomato	857
Tomato map	122
Mapped SNPs	29
Mapped indels	22
Mapped SSRs	6
Potato	729
Pepper	418
Coffee	442

^1^Homology based on single hits of UCD COS at e-20 with matching over 80% of the length of the SGN COS II

### The Size of Introns in Tomato

**Figure 1:**
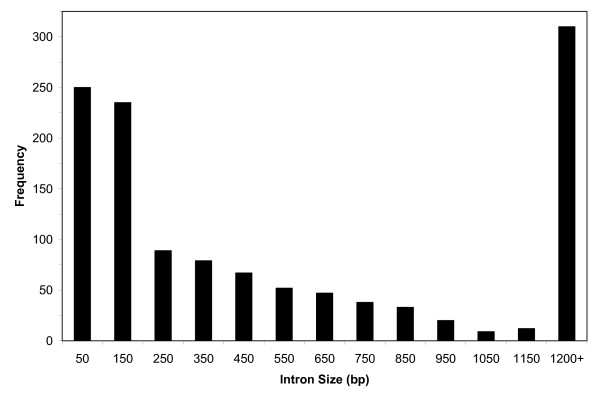
Distribution of intron sizes of 825 COS ESTs in tomato.

We designed primers spanning putative introns from 1,112 ESTs in the UCD COS. Greater than one primer set was designed for some contigs. Intron positions were predicted by comparing tomato COS ESTs to *Arabidopsis *protein sequence (See Materials and Methods) [18]. With these primers, 967 loci were amplified in 12 tomato lines (Table 2). Consequently, forward and reverse sequence across the resulting amplicons generated 1,241 predicted introns from 1102 unique contigs (1.33 contigs/locus) derived from 825 primer sets (Fig. 1). Greater than one contig/locus resulted when forward and reverse sequences did not overlap when primers spanned large introns. A total of 825 of the 967 loci yielded BLAST hits to the original COS EST database. The loci with poor BLAST results were due to the fact that the primers were located close to the intron (> 100 bp) resulting in the near lack of sequence to COS EST coding sequence. BLAST of genomic sequence data generated from PCR amplicons back to the corresponding EST allowed intron size to be estimated for each contig by subtracting the last position (3') of a query from the corresponding first position (5') of the next match. Intron positions were accurately predicted greater than 98% of the time based on differences between expected and predicted fragment sizes on agarose gels and sequence analysis. The experimentally estimated intron size ranged from 63 to 1469 bp. For 310 loci, the intervening region was either too large to form a contig from forward and reverse sequence pairs, or had no hits to ESTs. By assuming that the 310 pairs that did not assemble into a contig were comprised of introns that were at least 1,250 bp in length, the predicted average intron length in tomato would be 543 bp. These estimates are consistent with the limited information in tomato and suggest a two-fold intron size increase in tomato relative to *Arabidopsis *[14,19,20]. Furthermore, 263 EST sequences (23.8%) have more than one intron. A comparison of our intron sequences to the tomato repeat database (unirepeats.30.20060602,[21]) using BLAST indicated that there are few defined repeats in these tomato introns. Only 26 introns had homology to repeats in the database; one defined as a Long Terminal Repeat, another as DNA/Mutator and the rest are annotated as unknown repeats.

**Table 2:** Summary of lines used for sequencing.

Line	Market Class	Description	Use	Included in pool	Reference
M82	Processing	Inbred	Introgression line mapping	Pool 1	[31]
O 8245	Processing	Inbred, F1 parent	Color and lycopene QTL	Pool 1	[3, 32]
O 88119	Processing	Inbred, F1 parent	Disease resistance, IBC pop	Pool 1	[33]
Sun1642	Processing	Inbred	Fruit shape QTL mapping	Pool 1	[34]
Heinz 1706	Processing	Inbred	Donor for whole genome sequencing	Pool 2	[35, 36]
O 9242	Processing	Inbred, F1 parent	Color and lycopene QTL	Pool 2	[3, 8]
NC84173	Fresh Market	Inbred, F1 parent	Disease resistance QTL	Pool 2 & 3	[37]
Fla7600	Fresh Market	Inbred	Disease resistance, IBC pop	Pool 2 & 3	[33]
Ha7998	Breeding line	Inbred	Disease resistance, IBC pop	Pool 3	[33]
San Marzano	Heirloom			Pool 3	
PI114490	NA	*S. lycopersicum *var. cerasiformae	Disease resistance, IBC pop		[33]
LA1589	NA	*S. pimpinellifolium*	Mapping reference		[34]

### Diversity of Tomato Introns

Pooling of samples has been used to efficiently screen BAC libraries for presence of sequences, identify novel mutations through TILLING, estimate gene frequencies in populations, and identify variants for specific traits through bulk-segregant analysis. We extended this idea to pre-screen DNA sequences for polymorphism in germplasm. This approach is particularly efficient when screening narrow germplasm pools such as those found in the majority of agricultural crops, especially self-pollinated species such as tomato, which have gone through genetic bottlenecks during domestication and selection. Single-stranded conformation polymorphism was suggested as a tool for screening loci for polymorphism [22]. In our experience, this approach was only effective for amplicons smaller than 250 bp (data not shown), which is too small for efficient marker discovery in species with limited diversity and SNP density.

To develop an efficient strategy for SNP discovery in crops with narrow germplasm, we empirically determined that sequencing pools of four lines can effectively be employed to screen for polymorphisms (see Material and Methods). At first, we evaluated the variation among breeding lines and between species by sequencing across introns of 30 COS loci in 12 select tomato lines (See Table 2). This approach allowed us to refine the pooling strategy and establish a base line for polymorphism rate. This initial sequencing effort yielded 12 (40%) polymorphic loci. Amplicons for the remaining 937 loci were then sequenced in pools (See Materials and Methods) for polymorphism discovery. Of these loci, 309 (33%), showed a potential polymorphism within or between pools (Table 3). As the goal was to select for loci that were polymorphic in breeding germplasm, only *Sl *lines were included in the pools for screening. The pooled strategy had an accuracy rate of 98%, with 302 loci showing polymorphisms in at least one line when sequenced individually (Table 3, See Additional file 1). Therefore, we reduced the sequencing efforts three-fold by using the pooled genotypes strategy.

**Table 3:** Summary statistics for sequence database.

	**Number**	**Percentage**
Primers Tested	1268	
Amplified single copy	967	76
Tested in pooled sequencing	937	
Polymorphic in pools	309	33
Sequenced in lines	308	
Polymorphic	302	98

By using a pooling and re-sequencing strategy, we uncovered variation in ten domesticated breeding lines of *Sl*, one wild cherry tomato (*S. lycopersicum *var. cerasiformae, *Slc*) and the progenitor species, *S. pimpinellifolium *(*Sp*) across predicted introns in 302 of 967 ESTs (Tables 3 and 4). On average, we assayed a minimum of 985 high quality base pairs per locus (range 62–2262 bp) with an average sequence read length of 619 bp (see Additional file 1). Overall, 1,487 SNPs were identified, of which 579 from 162 loci were polymorphic in *Sl *breeding germplasm; 586 between *Slc *and other lines; and 1,121 between *Sp *and other lines (See Additional file 1). Interestingly, 402 SNPs were present within processing tomato lines and 168 within fresh market lines, but 400 differentiated the processing from fresh market classes indicating diversity among gene pools (Table 4, See Additional file 1). In the same samples, 282 indels were also detected in 153 contigs with 206 indels from 122 loci in breeding germplasm; 154 between *Slc *and other lines; and 152 between *Sp *and other lines. Fresh market and processing tomatoes differed by 45 indels (Table 2, See Additional file 1). Only 13 loci had indels and no SNPs. Polymorphisms due to SNPs or indels range from 8 to 24% between any two breeding lines (See Additional file 1). SSR signatures (2 to 4 nucleotide repeats) were identified in 44 loci of which 12 were polymorphic and 21 unique to the indel and SNP databases (See Additional file 1). Ten SSRs were scored as indels as well. Overall, 967 EST loci have sequence information within introns, of which 302 are polymorphic. All sequences are available at GenBank (ET165605 to ET166001)[23].

**Table 4:** Summary statistics for SNPs and indels in introns of 12 lines of tomato.

	**Loci with SNPs**	**Number of SNPs**	**% Loci with SNPs**	**SNPs/locus**	**Bases/SNP^1^**	**Loci with indels^2^**	**Number of indels**	**% Loci with indels**	**Indels/locus**	**Bases/indel^1^**
Total	302	1487	31.2	4.9	641	153	282	16	1.8	3378
*S. lycopersicum*	243	980	25.1	4.0	973	139	248	14	1.8	3841
Breeding lines^3^	162	579	16.7	3.6	1647	122	206	13	1.7	4624
US Fresh Market	53	168	5.5	3.2	5675	106	158	11	1.5	6028
Processing	121	402	12.5	3.3	2372	118	193	12	1.6	4935
var cerasiformae	161	586	16.6	3.6	1627	98	154	10	1.6	6185
*S. pimpinellifolium*	275	1121	28.4	4.1	851	98	152	10	1.6	6266

^1^Adjusted for % polymorphic loci and average contig length of 985 bp

^2^Thirteeen loci have indels and no SNPs

^3^Includes fresh market, processing and heirloom

Initial work [8] showed that SNP frequency is approximately 1/8500 bp when surveying coding sequence (ESTs) between TA496 and Rio Grande. The work was based on identifying SNPs between only two sequenced genotypes, and therefore is an underestimate of the variation across a larger set of lines. Within the 10 *Sl *breeding lines in the current project, 16.8% (162 of the 967 loci tested, See Additional file 1) of the loci were polymorphic with a SNP frequency of 1/1647 bp, a 5.2 fold increase over coding sequence reported [8]. Although, these datasets are not directly comparable due to both the number and actual lines and loci being compared, they are in line with those reported in the literature. Previous studies on a limited number of loci have reported a 2.7 to 5.3 fold [8,14,24] increase in SNP frequency in tomato introns vs. exons.

The results of our intron sequencing suggest that significant sequence diversity remains to be discovered in domesticated germplasm of tomato. These results appear to be corroborated by several studies in progress. Sequencing 48 COS for a set of 31 landrace *Sl *lines collected from three centers of diversity, found an average SNP rate of 1/149 bp across introns and exons with 60% of the SNPs having an allele frequency below 10% [25]. A second recent report showed that by randomly designing primers across ESTs (including exons, introns and untranslated regions) and sequencing 435 amplicons across 8 inbred lines, polymorphism ranged from 3% to 24% among *Sl *inbred lines to 63% between *Sl *inbred lines and *S. pennellii*, acc. LA716 [26]. The frequency of SNPs ranged from 10% of loci in processing lines, 11% in fresh market lines and 14% in cherry tomatoes (*Slc*). The frequency of minor alleles (allele with lowest frequency in germplasm surveyed) in tomato breeding germplasm was below 10% for 15/20 SNPs. The average frequency of minor alleles (SNPs) in our study was 18% across all the germplasm tested and 27% for breeding germplasm (See Additional file 1). The allele frequencies in our study may be higher as we pre-selected loci to be polymorphic in our germplasm and because our sampling was limited. SNPs with moderate allele frequencies (above 15%) are particularly useful as they are informative in relevant germplasm for breeding. Conversely, rare alleles can be particularly interesting if linked or are functional alleles for rare phenotypes for genetic studies and crop improvement. Although, we chose to sequence introns of ESTs from a COS, the frequency of polymorphisms is in a similar range as other reports [8,14,24]. It is not clear that COS genes would have less recombination than genes not conserved among species, especially in introns. Increasing the number and diversity of domesticated genotypes sequenced may increase the discovery of polymorphisms that can be applied to *Sl *breeding populations. The genotypes we screened (Table 2) represent genetic stocks and lines used in commercial processing and fresh market hybrids across the United States, Europe and Brazil.

In our data set, any given breeding line accounted for 11.3 to 23.8% of the polymorphism with 3–4 SNPs/locus in polymorphic loci. Considering that we prescreened 967 loci and sequenced 308 polymorphic loci (32%), we estimate that 3.8 to 7.9% of all loci differ in elite-by-elite crosses with the set of lines sampled. *Sp *was polymorphic in 91% of loci sequenced with a SNP frequency of 1/851 bp (Table 4, See Additional file 1). As only loci that were polymorphic in breeding lines were sequenced, this frequency for *Sp *is likely biased. Our estimate of polymorphism between *Sp *and *Sl *from the random set of 30 loci sequenced for all lines is 73%. As we only sampled two U.S. fresh market lines, our estimates for this class may have also been biased. By cross-referencing our dataset to current resources in the Asterid family, including the updated tomato unigenes and SGN COSII dataset [1,14], we have expanded the available COS in Solanaceae; provided intron sequence associated with ESTs; and diversity information relevant to loci that can form a core marker set for translational genomics research in the Solanaceae. Of the 967 loci screened, 122 including 29 SNP, 22 Indel, and 6 SSR loci have been mapped [1] (Table 1, See Additional file 1); the remaining loci are currently being mapped. These results further highlight that *Sl *has evolved considerably from *Sp *and the dilemma of application of resources built around wide crosses to elite-by-elite crosses. Our dataset represents the most in depth study of variation in domesticated tomato and one of its closest progenitor species to-date.

## Conclusion

Leveraging of genomic tools across species boundaries to assay variation in relevant germplasm is key to the application of these resources for crop improvement and comparative genomics. In species with limited variation due to bottlenecks from domestication and subsequent breeding, identifying sufficient polymorphism for genetic studies will require that sequence data cover more varieties and both coding and non-coding sequences. We have provided sequence data for 302 loci in 12 accessions relevant to breeding and genetic mapping in tomato These resources should enable SNP genotyping assays for high-throughput screening of germplasm for taxonomic, genetic mapping, functional analysis and breeding.

## Methods

### Plant materials

The tomato lines and species screened for this study were chosen based on their relevance to both tomato genetic research and to breeding programs. Both fresh market and processing germplasm are represented, with lines chosen to represent a diversity of environmental adaptations, market classes, parents of mapping populations, and genomic sequencing efforts. Inbred tomato lines were selected from the University of Florida and the University of North Carolina fresh market breeding programs, with selected lines of commercial interest (Table 2). Six processing lines were chosen to represent adaptation to arid (Sun1642 and M82) and humid environments (OH8245, OH88119, OH9242, and H1706); to genetic mapping (e.g. M82): and to the tomato genome sequencing project (H1706). The processing germplasm was also chosen, based on studies with molecular markers, to span sub-populations within the humid adapted germplasm (D. Francis, unpublished). Several of the processing lines are currently used as parents of hybrid varieties grown in the U.S., Brazil, and Europe. Accessions of the heirloom variety San Marzano, *S. lycopersicum *var. cerasiformae and a progenitor species, *S. pimpinellifolium *(Table 2) were selected to represent ancient varieties and closely related progenitor species. Thus the germplasm and specific polymorphisms are expected to be relevant to multiple genetic and plant improvement applications. All accessions represent inbred lines, thus should be homozygous for the majority of the loci.

### Defining intron position

Intron positions were predicted by comparing COS ESTs to the *Arabidopsis *protein database[18] which also defines the coordinates for intron positions in *Arabidopsis*. We used GenBank Parser [27] to extract of intron/exon information from *Arabidopsis *GenBank files and compile a protein dataset with known intron/exon positions. Individual COS ESTs were then queried using BLASTX against the protein database. Predicted intron positions in tomato ESTs, the best *Arabidopsis *hit and intron size in *Arabidopsis *are displayed graphically, using an Extended Mode add-on to the Contig Viewer Program [28].

### Primer design and screening

A database was established representing a COS of 1,704 tomato unigenes (1612 *Sl*, 29 *S. habrochaites *and 63 *S. pennellii*) from 113,932 ESTs [15]. From these, a single and longest EST was chosen to design primers. Using the tools developed for Compositae Genome database, the position of introns was first estimated using the procedures above. A set of 1,268 primers were designed to amplify across estimated intron sites with primers 50–100 bp from the intron. Amplification of primers was tested on a single line, M82.

Primers that successfully amplified a product were tested for polymorphism using sequencing in a series of three pools representing different degrees of diversity. The design has complementary pools representing each class (fresh market, processing, other) with one diverse line from an alternate class to maximize the chance of detecting a polymorphism within or among pools. Using a series of empirical tests with lines with known SNPs in ratios of 1:7, 1:5, 1:3 and 1:1, we determined that an unknown polymorphism can be reliably detected with sequencing with a 1:3 dilution. Pool 1 consisted of O 9242, FL7600, Ha7998, PI114490; Pool 2 included M82, O 8245, O 88119, NC84173 and; Pool 3 consisted of Sun1642, Heinz1706, O 9242, FL7600 (Table 2). DNA was extracted from each line and was combined in equi-molar concentrations.

For all sequencing reactions, forward and reverse primers were tailed with M13 sequences and sequenced using standard protocols for Sanger sequencing (Applied Biosystems, Foster City, CA) in forward and reverse directions using a ABI 3730 (Applied Biosystems, Foster City, CA). Trace files were trimmed with Phred options -trim_cutoff 0.02" which translates to Phred 17 score. [29]. Assembly was achieved with Phrap/Consed and options were set at " -retainduplicates and -forcelevel 5". These options were optimized to give the best trim and assembly parameters for calling SNPs. Stringent trim parameters are favored in this case to minimize the high number of false SNPs associated with poor sequence on the ends. Amplicon sizes were estimated and included in Additional file 1, Tables S2 and S3. To calculate a more accurate estimate than from gel electrophoresis, the sequenced contig(s) size was used as a minimum. When greater than one contig per locus was obtained as a result of unpredictably large introns, the forward and reverse contig sizes were added.

SNPs were first identified semi-manually using Polyphred as heterozygotes within pools or homozygous differences among pools. The line, M82, was used as reference to screen amplicons for single copy number. Amplicons with putative SNPs were then amplified in the individual 12 lines (Table 2) and sequenced as described above. Only SNPs showing both homozygous alleles were called. Data was extracted from Polyphred using custom scripts ([30] See Additional file 1). Similarly, data for indels were extracted from Polyphred. SSRs (di to tetra repeats) were extracted from all sequenced loci for M82, our reference line, and the various genotypes and reported for all sequenced individuals. The sequence database was analyzed for all known repeats for tomato [1]. All loci were cross-referenced to the SGN COSII for tomato, pepper, potato and coffee and associated maps [14].

## List of abbreviations

BAC: bacterial artificial chromosome

COS: conserved orthologous set

EST: expressed sequence tag

Indel: insertion/deletion

Sl: Solanum lycopersicum

*Slc: Solanum lycopersicum *var cerasiformae

SNP: single nucleotide polymorphism

Sp: Solanum pimpinellifolium

SSR: simple sequence repeat

## Authors' contributions

Allen Van Deynze. As PI, designed, supervised and analyzed the research with co-PIs.

Kevin Stoffel. Carried out and refined the approaches and preliminary analysis for the research.

C. Robin Buell. Carried out the analysis of SSRs

Jia Liu. Carried out the analysis of SSRs

Alexander Kozik. Bioinformatics analysis of source data and new sequence data

Esther van der Knaap. Designed research, discussed approaches and data and selection of germplasm

David Francis. Designed research, discussed approaches and data and selection of germplasm

All authors have read and approved the final manuscript

## Supplementary Material

additional file 1 **Table S1. **Cross-reference of 1704 UC Davis COS to Tomato0607 unigene build and SGN COS II[14]. **Table S2. **SNP genotypes for 12 lines in tomato and 302 UCDCOS ESTs. **Table S3**. Indel genotypes for 12 lines in tomato and 153 UCD COS ESTs. **Table S4**. Polymorphisms due to SNPs (a) and Indels (b) among lines. **Table S5**. SSR genotypes for 12 lines in tomato and 44 UCD COS ESTs. All raw sequences have been submitted to GenBank (#s ET165605 to ET166001). Click here for file
